# Posterior pole retinotomy for treatment of recurrent macular hole retinal detachment in highly myopic eyes: a pilot study

**DOI:** 10.1186/s12886-021-01973-9

**Published:** 2021-05-17

**Authors:** Xianggui Wang, Xuezhi Zhou, Ying Zhu, Huizhuo Xu

**Affiliations:** 1 Eye Center of Xiangya Hospital, Central South University, No. 87 Xiangya Road, KaiFu District, Changsha, 410008 Hunan China; 2 Hunan Key Laboratory of Ophthalmology, No. 87 Xiangya Road, KaiFu District, Changsha, 410008 Hunan China

**Keywords:** Macular hole retinal detachment, High myopia, Posterior pole retinotomy, Recurrent retinal detachment, Vitrectomy

## Abstract

**Background:**

To investigate the feasibility and efficacy of posterior pole retinotomy to treat recurrent macular hole retinal detachment (MHRD) in highly myopic patients.

**Methods:**

We performed a retrospective study and reviewed the medical records in our hospital between January 1, 2016 and December 31, 2018. Highly myopic patients who received posterior pole retinotomy with silicone oil tamponade for their recurrent MHRD after pars plana vitrectomy were included in the analysis. Postoperative retinal reattachment, best-corrected visual acuity (BCVA), macular hole closure, and complications were evaluated.

**Results:**

There were 11 patients (11 eyes) included in this study. All retinas were reattached. Silicone oil was successfully removed from all eyes 1.5–3 months after the surgery. Macular holes were completely closed in three eyes and remained flat open in eight eyes. The BCVA of all eyes improved significantly at 12 months after surgery (logarithm of the minimal angle of resolution, pre vs. postoperatively, 1.87 ± 0.44 vs. 1.15 ± 0.24, *P* < 0.05). None of the patients had complications such as endophthalmitis, fundus hemorrhage, retinal redetachment, and proliferative vitreoretinopathy.

**Conclusion:**

Posterior pole retinotomy is a safe and effective surgery to treat recurrent MHRD after pars plana vitrectomy in highly myopic patients.

## Background

One of the most serious complications in patients with high myopia is macular hole retinal detachment (MHRD), which can result in partial or complete vision loss [1]. Currently, pars plana vitrectomy (PPV) is the primary surgical choice to reattach the retina and close the macular hole in order to restore visual function. However, due to the long axial length, posterior scleral staphyloma, and severe chorioretinal atrophy, the outcome of PPV is not satisfactory even with posterior vitreous cortex clearing, epiretinal membrane and internal limiting membrane (ILM) peeling, and intravitreal gas or silicone oil tamponade [2]. It has been suggested that vitrectomy combined with macular buckling (L-type titanium plate, Ando plombe), scleral imbrications, or posterior scleral reinforcement could effectively shorten the ocular axis, alleviate posterior scleral staphyloma, and achieve retinal reattachment and macular hole closure [3–6]. However, these surgical procedures are complicated and can have certain adverse outcomes. Therefore, it is important to find new treatment options for myopic patients with recurrent MHRD after PPV.

Posterior pole retinotomy is a relatively new method of operation. Individual cases as well as small case series have reported similar procedures being used to repair large macular holes [7, 8]. We adopted this surgery in recurrent MHRD because it could be very useful to relieve the reverse vertical traction caused by posterior scleral staphyloma. The procedure is relatively simple with fewer complications, which makes it easier for wide clinical application. The purpose of the present study was to investigate the feasibility and efficacy of posterior pole retinotomy in the treatment of recurrent MRHD in patients with high myopia.

## Methods

### Study design

We performed a retrospective study and analyzed highly myopic patients who received posterior pole retinotomy for their recurrent MHRD at the Department of Ophthalmology in Xiangya Hospital between January 1, 2016 and December 31, 2018. The protocol for this study was approved by the Ethics Committee of Xiangya Hospital, Central South University, Hunan, China. The study adhered to the tenets of the Declaration of Helsinki.

### Study participants

Among highly myopic patients with recurrent MHRD, those who received posterior pole retinotomy were included in our study. These patients all met the following indications for posterior pole retinotomy: (1) axial length (AL) ≥ 28 mm with posterior scleral staphyloma; (2) initial MHRD treated with vitrectomy with ILM peeling and silicone oil tamponade; and (3) recurrence of MHRD after silicone oil removal combined with phacoemulsification and intraocular lens (IOL) implantation. In addition, patients with incomplete postoperative and follow-up records were excluded.

### Preoperative and postoperative evaluations

Routine eye examinations, including best-corrected visual acuity (BCVA) measurement, slit lamp examination, fundus examination, intraocular pressure (IOP) measurement by Goldmann applanation tonometry, color fundus photography, A/B-mode ultrasonography, and spectral-domain optical coherence tomography (SD-OCT, Carl Zeiss), were performed before and after surgery. Follow-up examinations were performed at 1 week, 1 month, 6 months, 12 months, and 18 months after surgery. The preoperative and postoperative BCVA, axial length (AL, measured by A-mode ultrasonography), and OCT images retrieved from the medical records were analyzed. Data from the last follow-up visit were used to determine the efficacy. SD-OCT images were used to evaluate the anatomical recovery of MHRD. Retinal reattachment was defined when the gap between the retinal neurocortical layer and the retinal pigment epithelium (RPE) layer disappeared.

### Surgical technique

All 11 patients underwent standard transconjunctival 3-port 23-gauge PPV by the same ophthalmologist. The operation was performed in the same way for all eyes. After the remaining posterior vitreous body was resected by triamcinolone acetonide (4 mg/mL)-assisted vitrectomy and the epiretinal membrane was removed with forceps, 0.0125% indocyanine green was used to stain the ILM. Additional ILM peeling was performed when needed to ensure that the peeling area reached the superior and inferior temporal vessel arcades. The arcuate retinotomy was performed at the temporal retina with a distance of 2PD to the macular hole, with a range extended from 90° to 120° after intraocular electrocoagulation. After the retina was flattened with the perfluorocarbon liquid, intraocular laser photocoagulation was performed at the retinal edge of the incision site. Finally, the perfluorocarbon liquid was drained through the gas–liquid exchange and the vitreous cavity was filled with silicone oil. Postoperatively, patients maintained a face-down position for a minimum of 8 h per day for at least 2 weeks.

At the follow-up visit 1.5 to 3 months after the surgery, silicone oil was extracted from the vitreous cavity through two conventional scleral 23-gauge puncture holes. The gas–liquid exchange was performed repeatedly to remove the residues of emulsified silicone oil in the vitreous cavity. The scleral opening was closed with a 7–0 absorbable suture.

### Statistical analysis

Statistical analysis was performed in SPSS (version 20.0, IBM Corp, Armonk, NY, USA). Visual acuity was converted into the logarithm of the minimal angle of resolution for statistical analysis. Finger counting and hand motion acuities were transformed to 2.0 and 3.0 logarithm of the minimal angle of resolution, respectively. Continuous variables, such as age, AL, follow-up time, and BCVA, are expressed as the mean (± standard deviation). Differences between preoperative and postoperative BCVA and AL were evaluated using paired *t* test. A *P* value < 0.05 was considered as statistically significant.

## Results

### General characteristics

We identified 112 highly myopic patients with recurrent MHRD at our hospital between January 1, 2016 and December 31, 2018. Among them, 11 patients (11 eyes, 4 men and 7 women) received posterior pole retinotomy for their recurrent MHRD. Their baseline clinical characteristics and the history of previous vitrectomies are shown in Table 1. Among the 11 eyes, six had one previous vitrectomy and the other five underwent two previous vitreoretinal surgeries. The preoperative and postoperative findings in these patients are shown in Table 2. The mean follow-up time was 13.9 (± 2.4) months (ranged between 12 and 18 months).

**Table 1:** Baseline characteristics and clinical history of the study participants

Patient	Age (years)	Gender	Eye	Days of decreased vision acuity	Times of previous vitrectomy	Methods of previous vitrectomy
1	40s	male	OD	7	2	PPV + ILM peeling + silicone oil
2	50s	female	OS	14	2	PPV + ILM peeling + silicone oil
3	60s	female	OS	14	1	PPV + ILM peeling + silicone oil
4	50s	male	OD	7	2	PPV + ILM peeling + silicone oil
5	50s	male	OD	18	1	PPV + ILM peeling + silicone oil
6	70s	female	OD	20	2	PPV + ILM peeling + silicone oil
7	60s	female	OS	7	1	PPV + ILM peeling + silicone oil
8	50s	female	OS	30	1	PPV + ILM peeling + silicone oil
9	60s	female	OD	45	2	PPV + ILM peeling + silicone oil
10	60s	male	OD	16	1	PPV + ILM peeling + silicone oil
11	60s	female	OS	60	1	PPV + ILM peeling + silicone oil

*OS* left eye, *OD* right eye

**Table 2:** Preoperative and postoperative findings in the study participants

Patient	AL (mm)	Time of SOR (months)	Preop BCVA LogMAR (Snellen equivalent)	OCT preop	Post-12 M BCVA LogMAR (Snellen equivalent)	OCT postop	Follow-up (months)
1	30.48	2	1.70 (20/1000)	MD + MH + ERM	1.10 (20/250)	Attached, MH	12
2	29.22	3	2.00 (FC/10 cm)	MD + MH	1.22 (20/333)	Attached, closed	18
3	29.40	1.5	2.00 (FC/20 cm)	MD + MH	1.10 (20/250)	Attached, MH	15
4	33.45	3	3.00 (HM/20 cm)	MD + MH	1.70 (20/1000)	Attached, MH	18
5	30.29	3	1.70 (20/1000)	MD + MH	1.00 (20/200)	Attached, MH	12
6	30.61	3	1.70 (20/1000)	MD + MH + ERM	0.82 (20/133)	Attached, closed	12
7	31.79	2	2.00(FC/20 cm)	MD + MH	1.22 (20/333)	Attached, MH	15
8	30.32	3	2.00 (FC/30 cm)	MD + MH	1.10 (20/250)	Attached, MH	15
9	32.35	3	1.40 (20/500)	MD + MH + ERM	1.00 (20/200)	Attached, MH	12
10	29.48	3	1.40 (20/500)	MD + MH	1.00 (20/200)	Attached, closed	12
11	32.44	1.5	1.70 (20/1000)	MD + MH	1.40 (20/500)	Attached, MH	12

*ERM* macular epiretinal membrane, *FC* finger count, *HM* hand move, *IOL* intraocular lens, *MD* macular detachment, *MH* macular hole, *Post-12 M* 12 months postoperatively; preop, preoperative, *SOR* silicone oil removal

### Reattachment of detached retina

After the removal of silicone oil, all 11 eyes achieved anatomical retinal reattachment. There was no retinal detachment occurring during the follow-up period. The retinal reattachment rate was 100%.

### Visual acuity outcomes

The postoperative visual acuities of all eyes were significantly improved compared with the preoperative visual acuities (Table 2). The mean preoperative BCVA LogMAR was 1.87 (± 0.44) for 11 eyes, which improved significantly to 1.15 (± 0.24) at the final visit after surgery (*P* < 0.05).

### Macular hole closure

Postoperative SD-OCT examinations showed that macular holes were completely closed in three eyes (27.3%) but remained flat open in eight eyes (72.7%).

### Complications

High IOP occurred in four eyes after surgery (36.4%). After treatment with IOP-lowering eye drops and removal of silicone oil at 1.5 to 2 months after surgery, high IOPs returned to normal in all patients. No major postoperative complications, such as endophthalmitis, vitreous hemorrhage, or proliferative vitreoretinopathy (PVR), occurred during the follow-up period.

### Representative cases

**Fig. 1:**
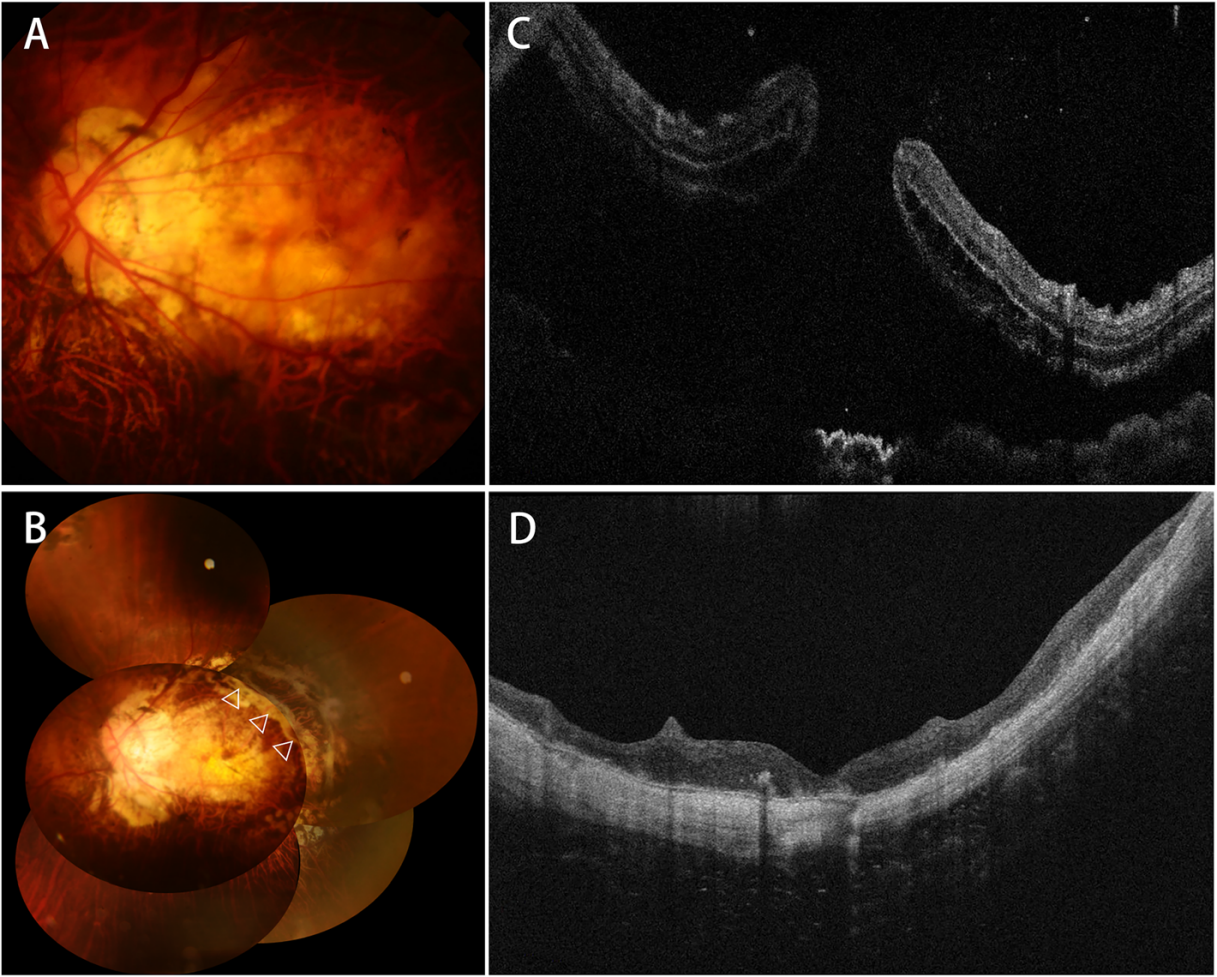
Color fundus photographs showing macular detachment in the left eye (**a**), the location (indicated by triangles) of posterior arcuate retinotomy, and retinal reattachment with no proliferative vitreoretiopathy at 6 months after the surgery (**b**). OCT shows a macular hole with retinal detachment before surgery (**c**) and macular hole closure and macular reattachment at 3 months after surgery (**d**)

Case 1 was a 52-year-old woman (Patient 2). Six months ago, she underwent two vitrectomies combined with silicone oil injections due to the myopic macular detachment of the left eye. Fourteen days ago, her vision started to decrease again. Fundus photograph and OCT revealed recurrent MHRD (Fig. 1a, c). After PPV with posterior pole retinotomy and silicon oil tamponade, macular reattachment was achieved and the macular hole was closed (Fig. 1b, d). Postoperative BCVA improved from FC/10 cm to 20/333 at the final visit 12 months after the surgery.

**Fig. 2:**
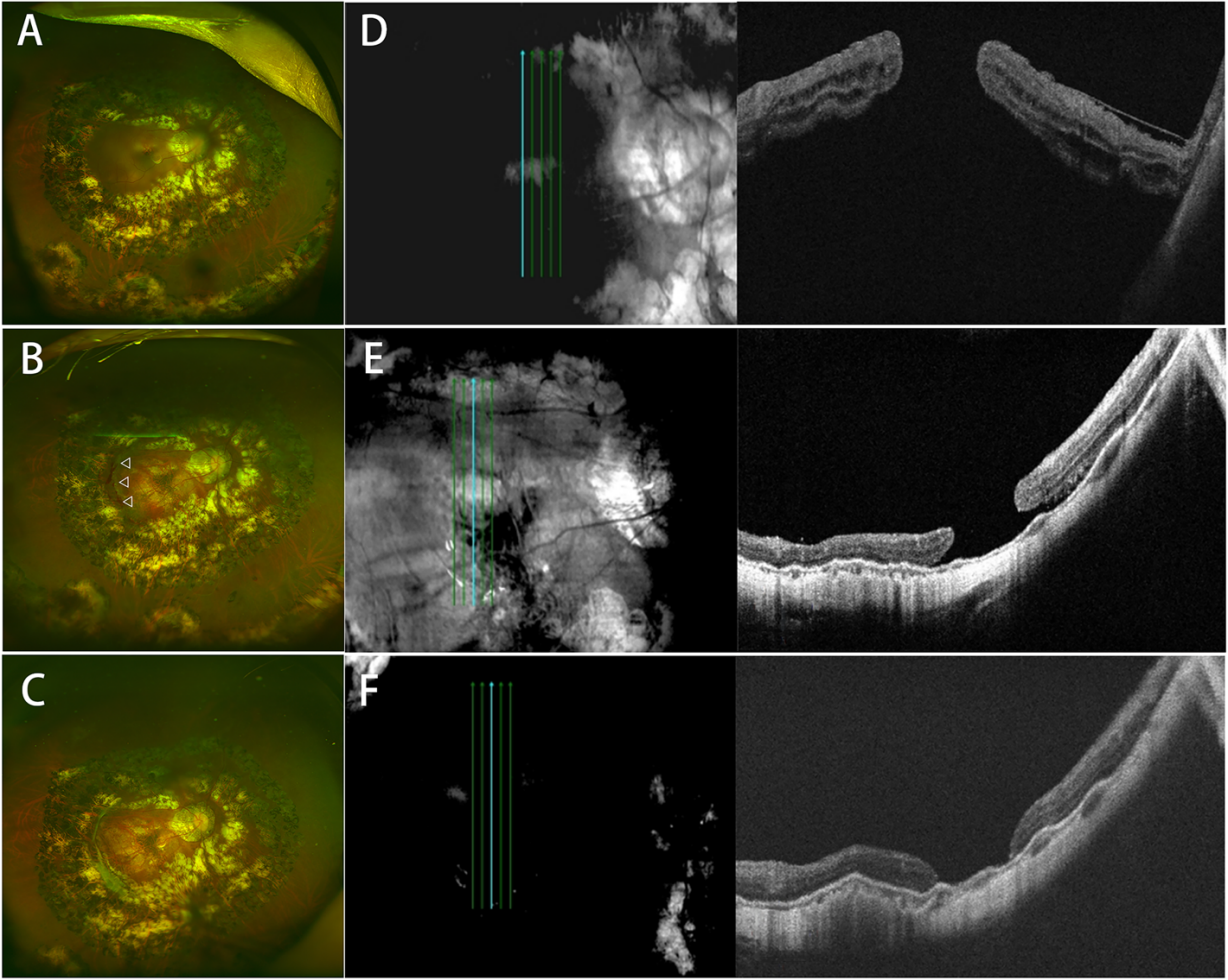
Color fundus photographs showing posterior pole with retinal detachment in the right eye (**a**), the location (indicated by triangles) of posterior arcuate retinotomy, retinal reattachment at 1 week after surgery (**b**), and retinal reattachment with no proliferative membrane at 3 months after surgery (**c**). OCT shows macular hole, epiretinal membrane, and macular detachment before surgery (**d**), and macular hole remained unclosed with macular reattachment at 1 week after surgery (**e**). Macular hole was flat open, and retina remained attached at 3 months following surgery (**f**)

Case 2 was a 69-year-old woman (Patient 9). Two years ago, she underwent two vitrectomies combined with silicone oil injections for myopic macular detachment of the right eye. This time, she was diagnosed as recurrent MHRD by OCT after presenting with decreased vision for 45 days (Fig. 2). After PPV with posterior pole retinotomy and silicon oil tamponade, macular reattachment was achieved, but the macular hole remained unclosed (Fig. 2f). Postoperative BCVA (Snellen equivalent) improved from 20/500 to 20/200 at the end of the 12-month follow-up period.

## Discussion

MHRD most often occurs in eyes with high myopia [9, 10]. MHRD caused by high myopia accounted for 0.5–5.0% and 9–21% of all retinal detachments worldwide and in China, respectively [11]. MHRD is one of the main reasons for visual impairment in highly myopic patients [12]. At the present time, the mechanism of MHRD in high myopia is unclear and involves many factors [13, 14], which include axial extension, thickening of the posterior vitreous cortex, abnormal retinal adhesion, long-term vertical and tangential traction of ILM or epiretinal membrane, reverse vertical traction of the posterior scleral staphyloma, rigid retinal arteriole, and severe choroidal atrophy in the macular area [15–19]. Vitrectomy with ILM peeling and silicone oil tamponade is currently the primary surgical method for treatment of MHRD in patients with high myopia [12, 14]. However, its treatment efficacy is controversial [2, 20]. Macular hole is difficult to close, and retinal detachment could recur after silicone oil removal or gas absorption [21]. Conventional PPV can clear the posterior vitreous cortex, strip the ILM or epiretinal membrane and remove the tangential or vertical traction factors of the macular area [22]. However, the axial extension and the reverse vertical traction caused by severe posterior scleral staphyloma still cannot be solved. Therefore, the outcomes of repeated PPV in patients with recurrent MHRD are not satisfactory, especially in patients with extremely high axial myopia. Some studies have presented favorable results with PPV combined with temporal scleral shortening, epimacular buckling, and posterior scleral reinforcement [23, 24]. The principle of these surgeries is to reinforce the weak area of the sclera at the posterior pole with special supporting materials in order to reduce the distance between the retina and the sclera [25]. Although this type of surgery has been shown to increase the success of retinal reattachment, it is complex and carries risks of multiple complications, including ischemic injury from the ocular tissue compression (especially the optic nerve or the choroid in the macular area), rejection of the supporting materials, astigmatism, submacular hemorrhage, malposition of buckle, and prolapse of orbital fat [26]. In addition, the exoplant materials used in macular buckle surgery are not commercially available in China, which limits the application of this surgery. It was reported that peripheral retinotomy, which is a common method to treat anterior proliferative vitreoretinopathy, could solve the problem of long axial length and retinal shortening [27]. However, it did not necessarily shorten the distance between the retina and the choroid in the posterior scleral staphyloma due to the convex shape. Moreover, the incision area is too large, which may cause severe damage to the retina and predispose to the postoperative proliferation. Therefore, an effective and safe treatment is still required to treat recurrent MHRD in highly myopic eyes.

In our study, 11 eyes with recurrent MHRD were successfully treated with posterior pole retinotomy. The retinal reattachment was 100% and no retinal detachment recurred during the follow-up period. Mean BCVA improved from 1.87 ± 0.44 to 1.15 ± 0.24 even though macular holes remained flat open in eight out of 11 eyes after surgery. Four eyes had high IOP postoperatively. No major complications were reported. In recurrent MHRD, since the posterior vitreous cortex, epimacular membrane (if present) and ILM have all been removed during previous operations, traction factors may not be the main reason for surgery failure. Therefore, we assumed the decreased retinal compliance caused by severe posterior scleral staphyloma might be the reason and attempted posterior pole relaxing retinotomy in our case series. Importantly, 45.45% (5/11) of patients in our study experienced recurrent RD after two vitrectomies combined with silicone oil injections, and they achieved retinal reattachment after posterior pole retinotomy. Though the macular hole closure rate was not high, retinal attachment was achieved in all patients in our study. This surgical approach could relieve the reverse vertical traction caused by the posterior scleral staphyloma and bring the posterior retina closer to the sclera in a simple way (as compared to macular buckling, scleral imbrications, or posterior scleral reinforcement). Our approach did not involve any supporting material, which avoided material-related complications, including astigmatism, ischemia, and rejection.

Posterior pole retinotomy might be a safe and effective surgical method for highly myopic patients with recurrent MHRD after PPV. However, our study had several limitations, including its retrospective design, small sample size, and short follow-up periods. Therefore, future large multi-center randomized controlled trials should be performed to determine the clinical value of posterior pole retinotomy in highly myopic patients with recurrent MHRD. Besides, retinotomy might cause disc changes and compromise retinal functions which needed to be monitored by visual field test, microperimetry and contrast sensitivity tests. High myopic patients already have constricted visual field and compromised visual quality, which makes it difficult to get objective appraisal for the possible damage caused by retinotomy. Considering this, we only recommend posterior pole retinotomy as the last resort for patients with recurrent MHRD after PPV.

## Conclusions

In summary, recurrent macular hole retinal detachment can occur in highly myopic eyes after pars plana vitrectomy. We performed a retrospective study and showed that posterior pole retinotomy is a safe and effective surgery to treat recurrent MHRD after pars plana vitrectomy in highly myopic patients. However, possible functional loss due to retinotomy needs to be monitored in the long term. Indications for posterior pole retinotomy should be carefully determined by surgeons.

## Data Availability

The datasets used and/or analyzed during the current study are available from the corresponding author on reasonable request.

## Abbreviations

AL: axial length
BCVA: best-corrected visual acuity
ERM: macular epiretinal membrane
FC: finger count
HM: hand move
IOL: intraocular lens
ILM: internal limiting membrane
IOP: intraocular pressure
MHRD: macular hole retinal detachment
MD: macular detachment
MH: macular hole
PPV: pars plana vitrectomy
PVR: proliferative vitreoretinopathy
postop: postoperative
preop: preoperative
SD-OCT: spectral-domain optical coherence tomography
SOR: silicone oil removal
